# Immunosuppressive Effect of Geniposide on Mitogen-Activated Protein Kinase Signalling Pathway and Their Cross-Talk in Fibroblast-Like Synoviocytes of Adjuvant Arthritis Rats

**DOI:** 10.3390/molecules23010091

**Published:** 2018-01-02

**Authors:** Feng Li, Miaomiao Dai, Hong Wu, Ran Deng, Jun Fu, Zhengrong Zhang, Li Dai, Wenyu Wang, Xuejing Dai, Xiang Zhan, Yan Wang

**Affiliations:** 1 College of Pharmacy, Anhui University of Chinese Medicine, Key Laboratory of Modernized Chinese, Medicine in Anhui Province, Hefei 230012, Anhui, China; wuhongprof@aliyun.com (F.L.); dengran94@aliyun.com (R.D.); fujun6866@163.com (J.F.); zhangzhengrong311@126.com (Z.Z.); daili6daisy@163.com (L.D.); 18605538039@163.com (W.W.); daixuejing@aliyun.com (X.D.); 18756935995@163.com (X.Z.); awangyan@163.com (Y.W.); 2 Hefei Anderson Pharmaceutical Co., Ltd., Hefei 230088, Anhui, China; fengli92@aliyun.com

**Keywords:** geniposide, adjuvant arthritis, MAPK signalling, cross-talk, fibroblast-like synoviocytes, rheumatoid arthritis

## Abstract

Geniposide (GE), an iridoid glycoside compound derived from *Gardenia jasminoides Ellis* fruit, is known to have anti-inflammatory and immunoregulatory activities. The aim of this study was to investigate the protective mechanism of GE in the regulation of the mitogen-activated protein kinase (MAPK) signalling pathway and the cross-talk among the MAPK signalling pathway in fibroblast-like synoviocytes (FLS) of adjuvant arthritis (AA) rats. AA was induced by injecting with Freund’s complete adjuvant. Male SD rats and FLS were subjected to treatment with GE (30, 60 and 120 mg/kg) in vivo from day 14 to 21 after immunization and GE (25, 50 and 100 μg/mL) in vitro, respectively. The proliferation of FLS was assessed by MTT. IL-4, IL-17, IFN-γ, and TGF-β1 were determined by ELISA. Key proteins in the MAPK signalling pathway were detected by Western blot. GE significantly reduced the proliferation of FLS, along with decreased IFN-γ and IL-17 and increased IL-4 and TGF-β1. In addition, GE decreased the expression of p-JNK, p-ERK1/2 and p-p38 in FLS of AA rats. Furthermore, disrupting one MAPK pathway inhibited the activation of other MAPK pathways, suggesting cross-talk among MAPK signalling. In vivo study, it was also observed that GE attenuated histopathologic changes in the synovial tissue of AA rats. Collectively, the mechanisms by which GE exerts anti-inflammatory and immunoregulatory effects may be related to the synergistic effect of JNK, ERK1/2 and p38. Targeting MAPK signalling may be a new therapeutic strategy in inflammatory/autoimmune diseases.

## 1. Introduction

Rheumatoid arthritis (RA) is a chronic, inflammatory, and systemic autoimmune disease, which is characterized by symmetric joint swelling and impaired small diarthrodial joints of the hands and feet [1,2]. Although the pathogenesis of RA leading to tissue inflammation and joint destruction involves different types of cells, cytokines, and other factors, abnormal proliferation of fibroblast-like synoviocytes (FLS) is a central event in the development of RA disease [3,4]. FLS secrete large amounts of matrix metalloproteinase (MMP) and stimulate the expression of inflammatory cytokines, such as interleukin (IL)-1, IL-6, IL-15 and tumour necrosis factor (TNF)-α, which could increase the production of adhesion molecules and cytokines from other cells, result in synovial inflammation and hyperplasia, and ultimately lead to joint destruction [5,6].

Geniposide (GE), an iridoid glycoside compound, is responsible for the pharmacological activities of *Gardenia jasminoides Ellis* fruit, which has long been used in traditional Chinese medicine [7]. One report has demonstrated that GE exhibited a protective effect on inflammation in lipopolysaccharide (LPS)-induced primary mouse macrophages in vitro by blocking the phosphorylation of p38MAPK, ERK1/2, JNK, and consequently inhibiting the secretion of TNF-α, IL-1β, and IL-6 [8]. Another study showed that GE inhibited the production of IL-6 and IL-8 in LPS-stimulated human umbilical vein endothelial cells by blocking p38MAPK and ERK1/2 pathways to exhibit anti-inflammatory and immunomodulatory effects [9]. These results suggested that the anti-inflammatory and immune activity of GE is closely related to MAPKs signalling. Therefore, therapeutic approaches aimed at modulating MAPKs signalling may have potential therapeutic advantages for inflammatory diseases.

Mitogen-activated protein kinase (MAPK) family has emerged as one of the most important enzymes in membrane-to-nucleus signalling transduction system. Activation of the MAPKs signal pathway occurs in three conservative enzymatic cascades: MAPK kinase kinase (MAPKKK/MEKK), MAPK kinase (MAPKK/MEK), and MAPK, which could act on transcription factor and regulate specific gene expression [10]. At present, MAPK subfamilies mainly include extracellular signal-regulated protein kinases (ERK1/2), c-Jun N-terminal protein kinase (JNK), and p38kinase [11]. It has been reported that the accurate position of these molecules in RA tissues was significantly different: the activation of ERK1/2, JNK, and p38MAPK positioning in the microvasculature, the infiltration of mononuclear cells, the synovial liners, and endothelial cells, respectively [12]. The ERK pathway is activated in response to signals from a variety of cell surface receptors. In contrast, p38MAPK and JNK pathways are primarily activated by cellular stress signals, such as pro-inflammatory cytokines, heat shock, and UV irradiation [13].

Our previous studies have demonstrated that GE exhibited an immunomodulatory effect on adjuvant arthritis (AA) rats. GE could inhibit the abnormal proliferation of peripheral blood lymphocytes (PBL) and FLS, repair the lower proliferation activity of mesenteric lymph node lymphocytes (MLNL), reduce the level of pro-inflammatory cytokines and increase anti-inflammatory cytokine production, and down-regulate the expression of p-JNK in PBL and MLNL, p-ERK1/2 in MLNL, and p-p38MAPK in PBL and FLS, thereby effectively relieving RA [14,15,16,17,18]. Based on our previous studies, in this experiment, we took effector cells (FLS) as the objective, to investigate the intervention of GE on MAPK signalling pathways and the cross-talk among the MAPK signalling in FLS of AA rats. It may be an important guiding significance for the pathogenesis of RA, the treatment mechanism of GE and the development of new drugs for the treatment of RA.

## 2. Results 

### 2.1. Evaluation of Arthritis on AA Rats

**Figure 1:**
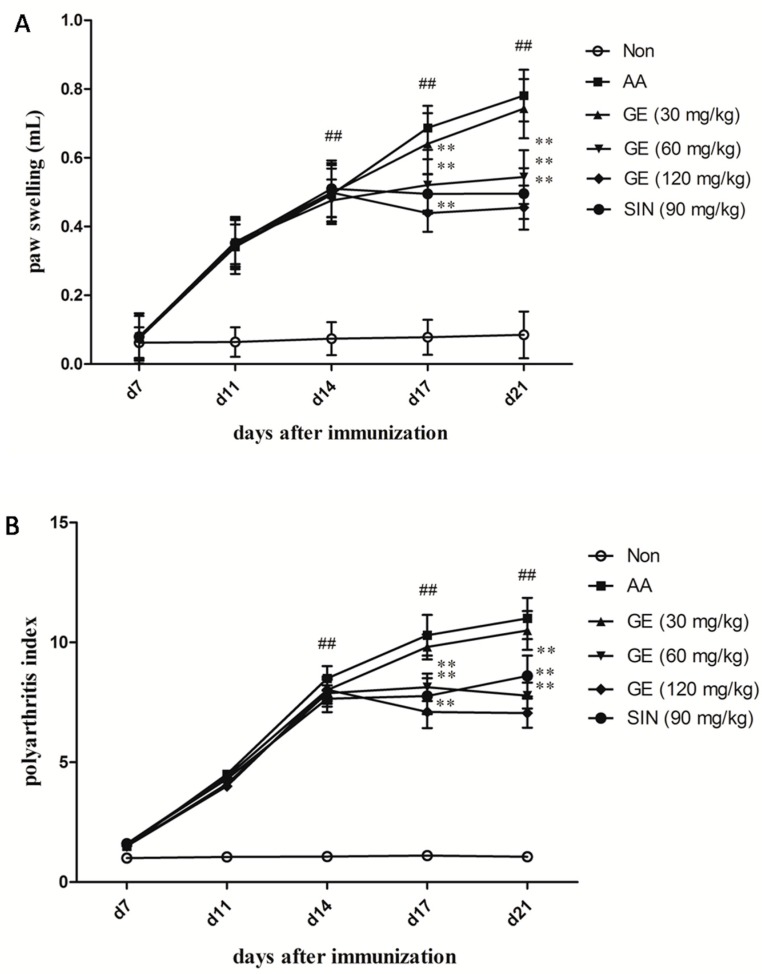
Effects of GE on secondary arthritis in AA rats. Rats were immunized by a single subcutaneous injection into the left hind metatarsal footpad with 100 μL of FCA for each rat. GE (30, 60, and 120 mg/kg) and SIN (90 mg/kg) were given intragastrically to AA rats from day 14 to 21 after immunization. The paw swelling and polyarthritis index were evaluated as described in Materials and Methods. (**A**) Paw swelling and (**B**) Polyarthritis index. Values are presented as means ± SD (n = 10). ^##^ *p* < 0.01 vs. the non-immunized group; ** *p* < 0.01 vs. the AA model.

The onset and peak of the inflammatory response occurred on day 14 and days 18–21 after immunization. The right hind paw swelling and polyarthritis index (secondary arthritis) were significantly increased in AA rats compared with non-immunized (Non) rats (*p* < 0.01). GE (60 and 120 mg/kg) apparently suppressed the hind paw swelling and polyarthritis index from day 14 to day 21 after immunization (*p* < 0.01), while there was no significant reduction in polyarthritis index by treatment with GE at 30 mg/kg (*p* > 0.05). The efficacy of sinomenine hydrochloride (SIN) (90 mg/kg) was similar to that of GE (60 mg/kg) (*p* > 0.05) (Figure 1).

### 2.2. The Effects of GE on the Joint Histopathology of AA Rats

**Figure 2:**
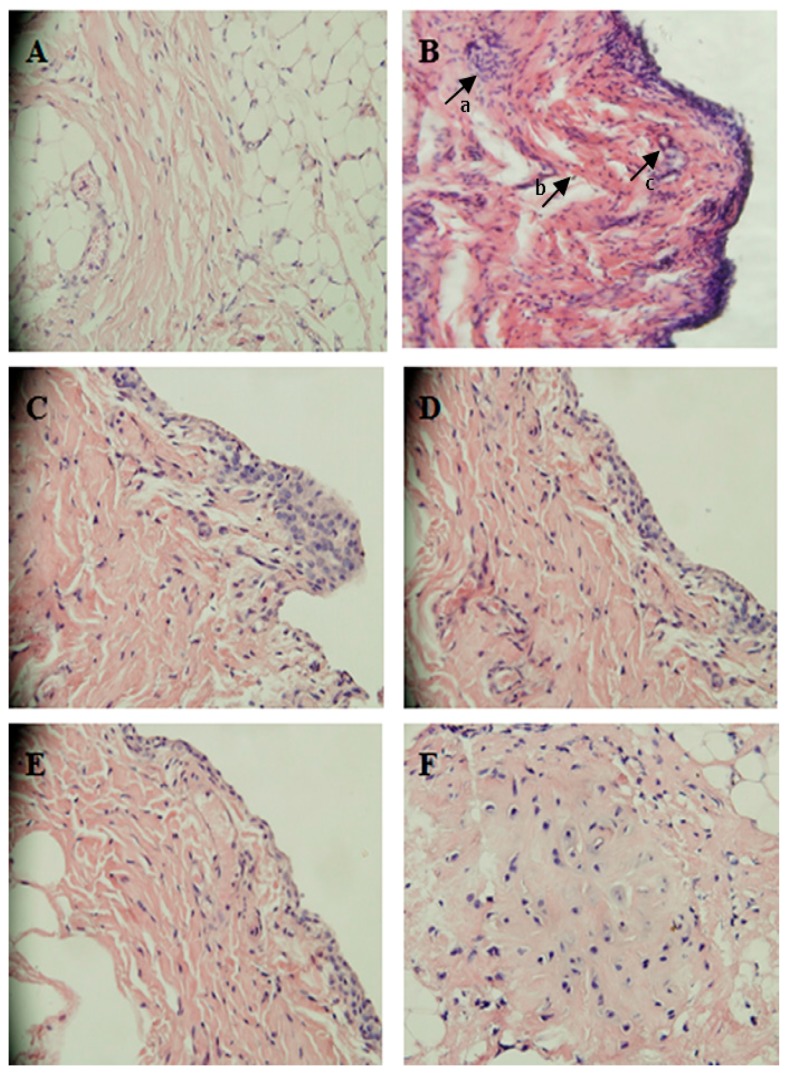
Histopathologic examination of synoviumin in AA rats (HE, 200×). (**A**) Non-immunized rats: no synovium hyperplasia and inflammatory cells infiltration were observed; (**B**) AA rats: inflammatory cells infiltration, synovium hyperplasia, and pannus formation were markedly observed. Arrows a, b, and c represent inflammatory cell infiltration, synovium hyperplasia and pannus formation, respectively; (**C**) AA rats treated with GE (30 mg/kg): a clear synovium hyperplasia and inflammatory cell infiltration; (**D**) AA rats treated with GE (60 mg/kg): synovium hyperplasia and inflammatory cells infiltration were obviously reduced; (**E**) AA rats treated with GE (120 mg/kg): little synovium hyperplasia were observed; (**F**) AA rats treated with SIN (90 mg/kg): fewer synovium hyperplasia and inflammatory cell infiltration were observed.

The typical characteristics of RA include synovial hyperplasia and joint destruction. Histopathological features were observed on day 21 after immunization. The histopathological analysis of joints showed in the AA group, synovial hyperplasia, inflammatory cell infiltration, and pannus formation were markedly observed compared with the Non group (Figure 2B). These abnormalities were evidently alleviated in AA rats after treatment with GE (60 and 120 mg/kg) and SIN (90 mg/kg) (Figure 2).

### 2.3. The Effect of GE on FLS Viability

**Figure 3:**
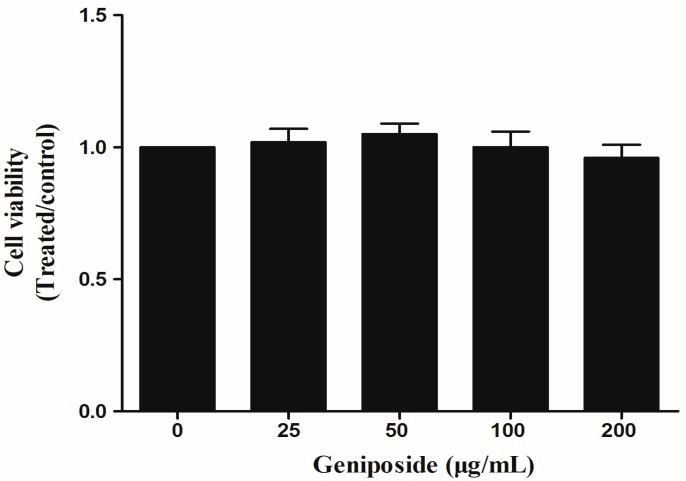
The effect of GE on the viability of FLS. FLS were cultured in triplicate in a concentration of 1 × 10^6^ cells/mL in 100 μL DMEM medium containing 10% FBS. Cell viability was measured with CCK8 assays in FLS after 48 h of treatment with GE. Values are presented as means ± SD (n = 3).

CCK8 assays showed that treatment with GE (25, 50, 100, and 200 μg/mL) for 48 h had no effects on FLS viability relative to control cells (*p* > 0.05) (Figure 3). The results showed that the inhibitory effect observed in the proliferation and inflammation was not caused by cytotoxic effects.

### 2.4. Effects of GE on the Proliferation of FLS

#### 2.4.1. Effects of GE on the Proliferation of FLS In Vivo

**Figure 4:**
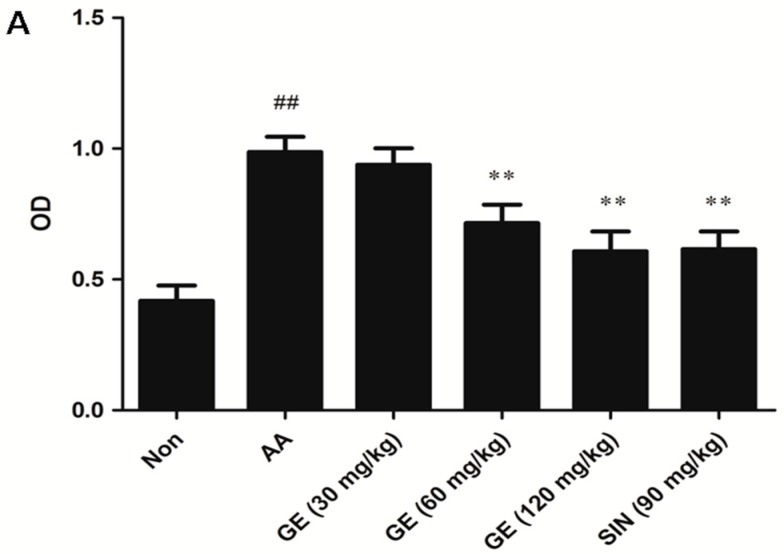

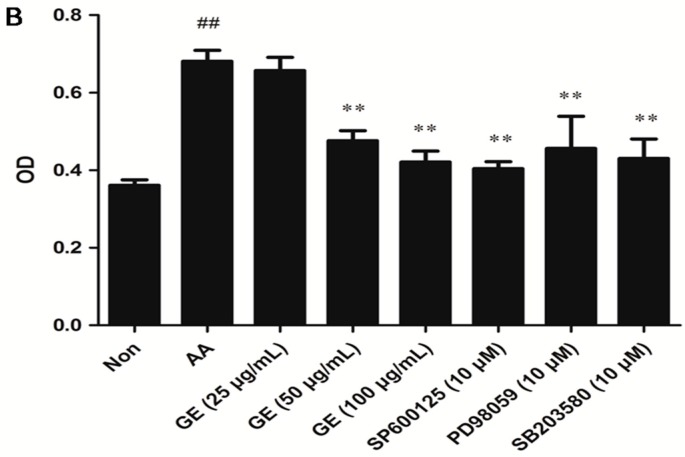
Effects of GE on the proliferation of FLS. Synovial tissues of AA rats were removed under sterile conditions. FLS were cultured as described previously. Then, the cells were resuspended in triplicate in a concentration of 1 × 10^6^ cells/mL in 100 μL DMEM containing 10% FBS. The cells were stimulated with LPS (10 mg/L) for 48 h at 37 °C and 5% CO_2_. FLS proliferation was determined by MTT assay. (**A**) The proliferation of FLS in vivo; (**B**) The proliferation of FLS in vitro with SP600125, PD98059, and SB203580 (10 μM), respectively. Values are presented as means ± SD (n = 3). ^##^ *p* < 0.01 vs. the non-immunized group; ** *p* < 0.01 vs. the AA model.

Hyperproliferation of FLS is a central event in the development of RA disease. Thus, we examined the effects of GE on AA FLS proliferation in this study. Compared with the Non group, the proliferation of FLS in AA group increased evidently (*p* < 0.01). GE (60 and 120 mg/kg) apparently suppressed FLS proliferation in AA rats (*p* < 0.01), SIN (90 mg/kg) produced similar effects as GE (120 mg/kg) (*p* > 0.05) (Figure 4A).

#### 2.4.2. Effects of GE on the Proliferation of FLS In Vitro

As shown in Figure 4B, compared with Non group, the proliferation of FLS in the AA group increased significantly (*p* < 0.01). GE (50 and 100 μg/mL) significantly inhibited the cell proliferation (*p* < 0.01). SP600125, PD98059, and SB203580 (10 μM) also had a significant inhibitory effect on cell proliferation (*p* < 0.01). 

### 2.5. Effects of GE on Production of IFN-γ, IL-17, IL-4, and TGF-β1 in FLS

#### 2.5.1. Effects of GE on Production of IFN-γ, IL-17, IL-4, and TGF-β1 in FLS In Vivo

**Figure 5:**
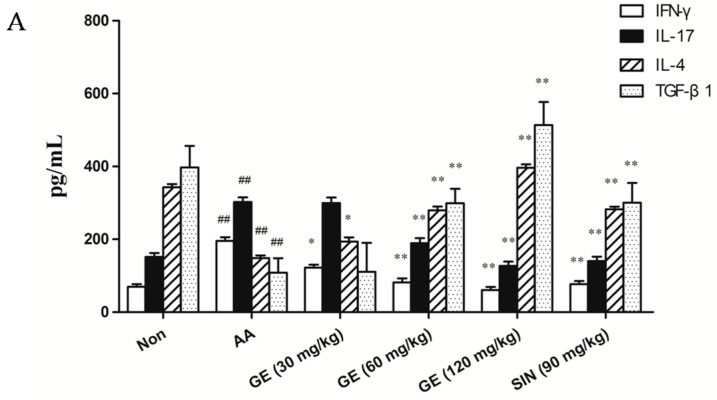

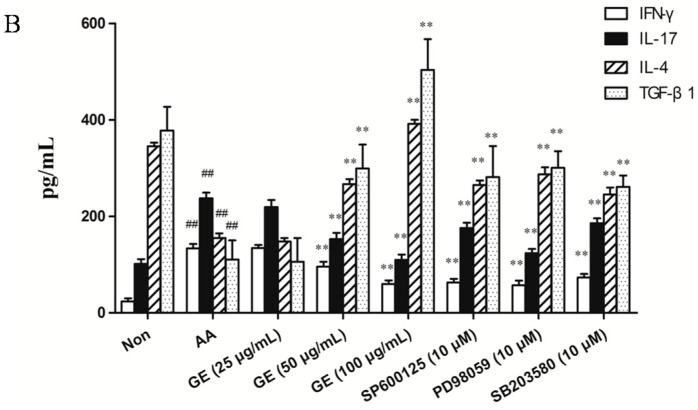
Effects of GE on production of IFN-γ, IL-17, IL-4, and TGF-β1 in FLS of AA rats. FLS were collected on day 21 after immunization. Levels of IFN-γ, IL-17, IL-4, and TGF-β1 were measured by ELISA. (**A**) Levels of IFN-γ, IL-17, IL-4, and TGF-β1 in FLS in vivo; (**B**) Levels of IFN-γ, IL-17, IL-4, and TGF-β1 in FLS in vitro with SP600125, PD98059, and SB203580 (10 μM), respectively. Values are presented as means ± SD (n = 3). ^##^ *p* < 0.01 vs. the non-immunized group; * *p* < 0.01, ** *p* < 0.01 vs. the AA model.

The levels of pro-inflammatory cytokines (IFN-γ and IL-17) were increased and the levels of anti-inflammatory cytokines (IL-4 and TGF-β1) were decreased in FLS of AA rats when compared with the Non group (*p* < 0.01). GE (60 and 120 mg/kg) significantly declined the levels of IFN-γ and IL-17, and elevated that of IL-4 and TGF-β1 in FLS of AA rats (*p* < 0.01). SIN (90 mg/kg) produced similar effects to those caused by GE (60 mg/kg) (*p* > 0.05) (Figure 5A).

#### 2.5.2. Effects of GE on Production of IFN-γ, IL-17, IL-4, and TGF-β1 in FLS In Vitro

Compared with the Non group, production of IFN-γ and IL-17 were increased, while that of IL-4 and TGF-β1 was decreased in FLS (*p* < 0.01). GE (50 and 100 μg/mL) obviously declined the levels of IFN-γ and IL-17, and elevated that of IL-4 and TGF-β1 in FLS of AA rats (*p* < 0.01). SP600125, PD98059, and SB203580 (10 μM), also observed, had the effect of decreasing the content of IFN-γ and IL-17, and increasing the content of IL-4 and TGF-β1 (*p* < 0.01) (Figure 5B).

### 2.6. Effects of GE on MAPK Pathways and Cross-Talk among MAPK Pathways in FLS

#### 2.6.1. Effects of GE on MAPK Pathways and Cross-Talk among MAPK Pathways in FLS In Vivo

**Figure 6:**
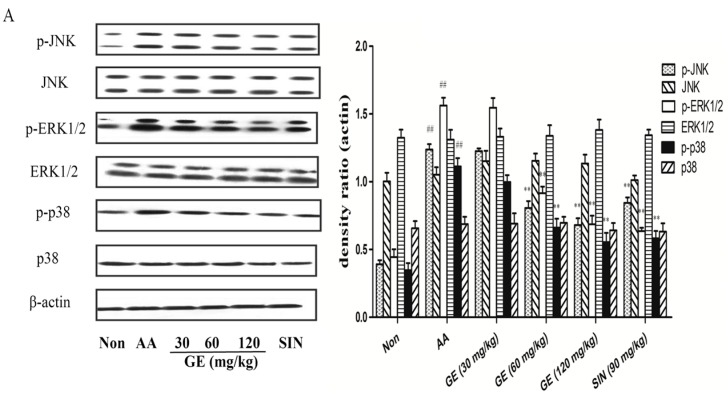

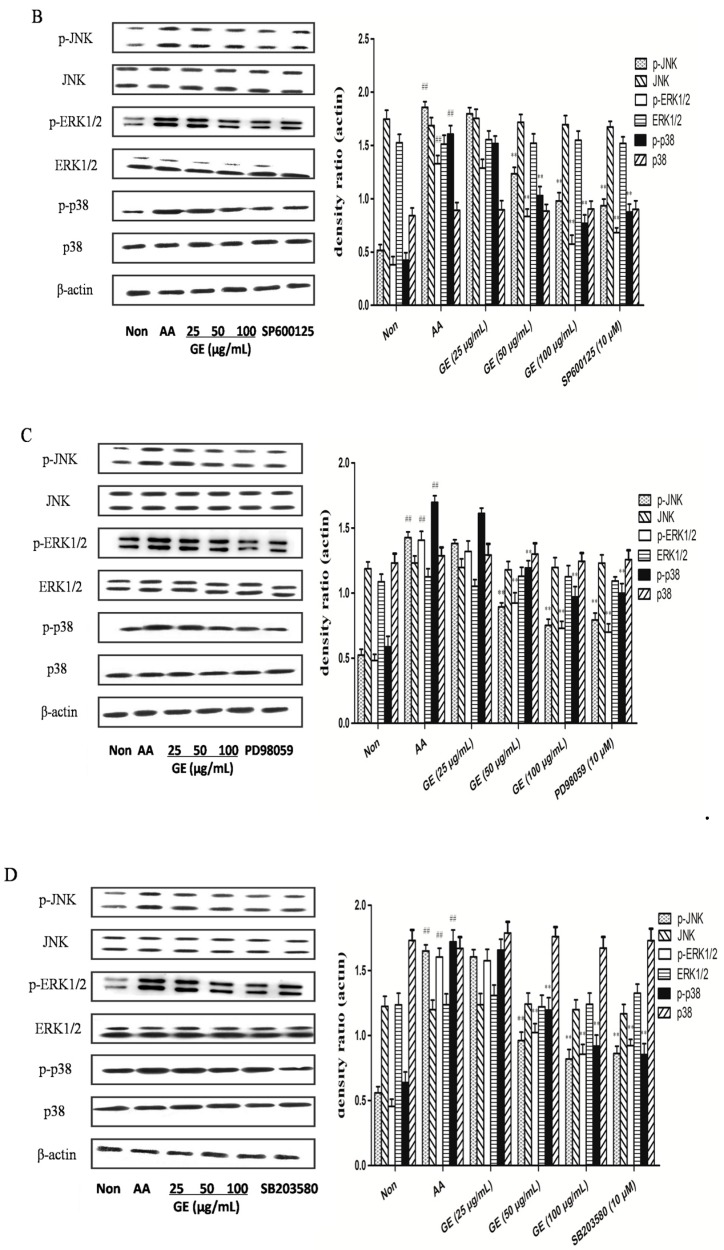
Effects of GE on MAPK pathway and cross-talk among MAPK pathways in FLS of AA rats. The proteins expression of MAPK signalling pathway were assessed by western blot on day 21 after immunization, as described in Materials and Methods. (**A**) Western blot for JNK, p-JNK, ERK1/2, p-ERK1/2, p38, and p-p38, and quantitative evaluation of these proteins in FLS in vivo; (**B**–**D**) Western blot for JNK, p-JNK, ERK1/2, p-ERK1/2, p38, and p-p38, and quantitative evaluation of these proteins in FLS in vitro with SP600125, PD98059, and SB203580 (10 μM), respectively. Values are presented as means ± SD (n = 3). ^##^ *p* < 0.01 vs. the non-immunized group; ** *p* < 0.01 vs. the AA model.

The protein expression of β-actin, JNK, p-JNK, ERK1/2, p-ERK1/2, p38, and p-p38 of FLS in AA rats was estimated on day 21 after immunization. It was found that the expression of p-JNK, p-ERK1/2, and p-p38 was heightened in of AA rats when compared with the Non group (*p* < 0.01). GE (60 and 120 mg/kg) markedly decreased that of p-JNK, p-ERK1/2, and p-p38 (*p* < 0.01). SIN (90 mg/kg), also observed, had the similar effect to those caused by GE (120 mg/kg) (*p* > 0.05) (Figure 6A).

#### 2.6.2. Effects of GE on MAPK Pathways and Cross-Talk among MAPK Pathways in FLS In Vitro

In in vitro experiments, GE (50 and 100 μg/mL) markedly decreased that of p-JNK, p-ERK1/2, and p-p38 (*p* < 0.01), while the protein levels of non-phosphorylated MAPK isoforms did not vary significantly between groups (Figure 6B–D). In addition, JNK inhibitor (SP600125, 10 μM) inhibited JNK activity while suppressing ERK and p38 activation. ERK1/2 inhibitor (PD98059, 10 μM) inhibited the ERK pathway while also suppressing JNK and p38 activation. Similarly, p38MAPK inhibitor (SB203580, 10 μM) interfered with the p38 pathway, and also attenuated JNK and ERK activation in FLS of AA rats. 

## 3. Discussion 

RA is a chronic, progressive and inflammatory disease, which is characterized by cellular and humoral responses. AA is the most commonly experimental model for human RA because of its similarity to human RA in clinical and pathological features. As shown in Figure 1, GE (60 and 120 mg/kg) significantly relieved the secondary hind paw swelling and polyarthritis index, indicating a protective effect on FCA-induced injury in vivo, while GE at 30 mg/kg had no significant inhibitory effect on the secondary hind paw swelling and polyarthritis index in AA rats, which is probably due to an inadequate dose and the short treatment duration of GE. In addition, Figure 2 showed that GE could improve the pathological state of synovium in AA rats. These results indicated that GE could alleviate the degree of secondary inflammatory response in AA rats with FCA-induced injury. The efficacy was similar to that of SIN (90 mg/kg).

Sinomenine hydrochloride (SIN), an active alkaloid isolated from the roots of a medicinal plant *Sinomenium acutum*, a commonly used herb in traditional Chinese medicine, and SIN has been frequently used in the clinic to treat RA and other inflammatory disorders because of its anti-inflammatory and immunosuppressive activities. Now, SIN has been developed as a market drug in China, named, Zhengqing Fengtong Ning [19]. In this study, it was used to observe the efficacy of AA rat, as the positive control drug. Although SIN has a significant effect in the treatment of RA, there is no relevant literature research about its regulating mechanism of MAPK pathways in FLS. Therefore, in this study, SIN is only used as a positive drug in observing the therapeutic effect of GE on AA rats to indicate the effects of GE for the treatment of RA.

A major feature of RA is synovial membrane proliferation. Although infiltration of inflammatory cells appears in the lining layer of synovial tissue, the excessive proliferation of synovial cells is the main reason for the hyperplasia of synovial tissue. The lining layer of synovial tissue consisted of A-type macrophage-like synoviocyte (MLS) and B-type FLS. Under normal circumstances, FLS play an important role in maintaining joint homeostasis. Mature FLS could secrete a large of long chain poly hyaluronic acid, which has lubrication and immunomodulatory functions [20]. MLS is a terminally differentiated cell and does not have the proliferation ability in vitro. Furthermore, FLS is dominated in hyperplastic synovial cells, so FLS was chosen for the study. The results showed that the proliferation of FLS induced by LPS in AA rats was significantly higher than the normal group, while both GE (60 and 120 mg/kg) in vivo and GE (50 and 100 μg/mL) in vitro significantly decreased the proliferation of FLS, suggesting an inhibitory effect of GE on the abnormal proliferation of synoviocytes in AA rats. Furthermore, the JNK inhibitor group (SP600125, 10 μM), ERK1/2 inhibitor group (PD98059, 10 μM), and p38MAPK inhibitor group (SB203580, 10 μM) had similar effects as GE, indicating that GE inhibited the proliferation of FLS in AA rats probably by blocking the activation of the MAPK pathway. ERK1/2, JNK, and p38MAPK pathways are three different MAPK signalling pathways and play an important role in RA. In order to investigate the intervention of GE on MAPK signalling pathways and the cross-talk among the MAPK signalling in FLS of AA rats, PD98059 (ERK1/2 inhibitor), SP600125 (JNK inhibitor), and SB203580 (p38MAPK inhibitor) were chosen as positive control drugs to indicate the effects of GE on the MAPK signalling pathway of FLS in vitro.

In this experiment, the levels of IFN-γ and IL-17 were significantly increased, while the production of IL-4 and TGF-β1 was markedly decreased in FLS of AA rats. Both GE (60 and 120 mg/kg) in vivo and GE (50 and 100 μg/mL) in vitro could obviously reduced the elevated level of IFN-γ and IL-17, and rapidly increased the lower level of IL-4 and TGF-β1, which ultimately inhibited the secretion of inflammatory cytokines IFN-γ and IL-17, and corrected the immune balance of inflammatory/anti-inflammatory cytokine, so as to play a persistent suppression effect on AA rats. Besides, JNK inhibitor group (SP600125, 10 μM), ERK1/2 inhibitor group (PD98059, 10 μM), and p38MAPK inhibitor group (SB203580, 10 μM) had similar effects as GE, indicating that GE inhibited the secretion of inflammatory cytokines in FLS of AA rats maybe by blocking the activation of MAPK pathway.

To explore the protective mechanism for the role of GE in AA rats, we analysed the activation of MAPK signalling pathways. Although anti-inflammatory properties of GE have been identified, the mechanism of action has not yet been fully elucidated. In this experiment, we explored that the MAPK signalling pathway is involved in inflammatory injury of FLS in AA rats and the anti-inflammatory effects of GE are related to the inhibition of phosphorylated proteins in MAPK signalling pathway. The results showed that the expression of p-ERK1/2, p-JNK, and p-p38 was significantly up-regulated in FLS of AA rats, suggesting that MAPK signal pathway was activated. Both GE (60 and 120 mg/kg) in vivo and GE (50 and 100 μg/mL) in vitro markedly down-regulated the levels of p-ERK1/2, p-JNK, and p-p38 in FLS of AA rats, while that of total proteins had no change.

Various MAPK cascades (ERK1/2, JNK, p38, and ERK5) are often described in previous literature as linear cascades. However, there have been increasing signs of cross-talk between the various cascades in recent years. For example, growth factor-stimulated p38 could induce the migration of epithelial cells, while ERK1/2 activation could induce the proliferation of epithelial cells. The cross-talk between p38 and ERK1/2 coordinate the dynamics of corneal wound healing [21]. Shen et al. [22] found that the expression of active mixed lineage kinase (MLK) 3 in serum-deprived COS-7 cells almost completely attenuates ERK activation in response to epidermal growth factor (EGF) and phorbol ester (PMA), this attenuation depends on MLK3 activity. JNK inhibitor I and JNK inhibitor II completely reverse MLK3-induced ERK inhibition. In addition, JNK inhibitor I reverse MLK3-induced ERK inhibition without affecting the phosphorylation of MEK-1 induced by MLK3, indicating that the inhibition of ERK activation is independent of MEK activation, but requires JNK activity. These results demonstrated that sustained activation of JNK signalling could uncouple ERK activation from MEK-1 in a manner requiring c-Jun-mediated gene transcription. Additionally, another report has suggested opposite roles for JNK and p38MAPK on cardiomyocytes, in which the activation of p38MAPK increased contractile proteins expression and protected cardiomyocytes from death, while the activation of JNK suppressed the effect of p38MAPK and induced cardiomyocytes apoptosis [23]. Disruption of a MAPK pathway could influence the TGF-β1-induced activation of other MAPK pathways, suggesting cross-talk among MAPK signalling pathways [24].

In our in vitro experiment, inhibition of JNK activity with JNK inhibitor (SP600125) suppressed ERK and p38 activation. Inhibition of the ERK pathway with ERK inhibitor (PD98059) also restrained JNK and p38 activation. Similarly, blockade of the p38 pathway with p38 inhibitor (SB203580) attenuated JNK and ERK activation in FLS of AA rats. These results suggested that the three MAPK pathways communicate with each other negatively. Further work will be required to elucidate the detailed mechanisms by which inhibition of one MAPK pathway regulates the activities of other MAPK pathways in AA.

## 4. Materials and Methods

### 4.1. Animals

Sprague-Dawley (SD) rats (male, 180 ± 20 g. Grade Ⅱ, certificate no. 011) were purchased from the Animal Department of Anhui University of Chinese Medicine (Hefei, Anhui Province, China). All animals were housed under specific pathogen-free conditions with a 12-h light/dark cycle and allowed food and water ad libitum. Laboratory temperature was 23 ± 2 °C and relative humidity was 50–60%. All Animal experiments were performed in accordance with protocols approved by the Ethics Review Committee for Animal Experimentation of Anhui University of Chinese Medicine (project identification code: 2015-047 and approval date: 16 March 2015).

### 4.2. Reagents

**Figure 7:**
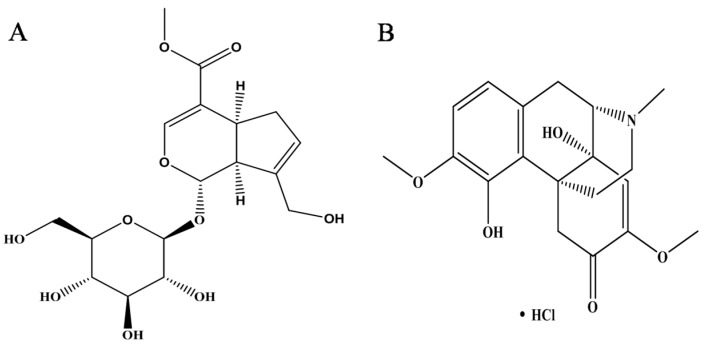
Chemical structure of Geniposide and Sinomenine hydrochloride. (**A**) Geniposide: therapeutic drugs; (**B**) Sinomenine hydrochloride: positive control drug.

GE (Figure 7A) and SIN (Figure 7B) (purity > 98.0%) were provided by the National Institute for the Control of Pharmaceutical and Biological Products (Beijing, China). The enzyme-linked immunosorbent assay (ELISA) kits for IFN-γ, IL-17, IL-4 and TGF-β1 were supplied from Elabscience Biotechnology Co., Ltd. (Wuhan, China). Rabbit mAb JNK, p-JNK, ERK1/2, p-ERK1/2, p38, p-p38, and β-actin were obtained from Bioworld Technology Inc. (Nanjing, China). HRP-conjugated goat anti-rabbit antibodies were purchased from ZSGB-BIO (Beijing, China). JNK inhibitor (SP600125), ERK inhibitor (PD98059), and p38 inhibitor (SB203580) were obtained from Calbiochem-Novabio chem Corp. (San Diego, CA, USA). Lipopolysaccharide (LPS), Cell counting kit-8 (CCK8) and 3-(4,5-dimethylthiazol-2-yl)-2,5-diphenyltetrazolium bromide (MTT) were offered from Sigma Chemical Co. (St. Louis, MO, USA); Dulbecco’s modified Eagle’s medium (DMEM) was purchased from Thermo Scientific Co. (Waltham, MA, USA). All other chemicals used in this study were of analytical grade and were obtained from commercial sources.

### 4.3. Experimental Design

The AA model was induced in SD rats by subcutaneous injection into the left hind metatarsal footpad with 100 μL of Freund’s complete adjuvant (FCA). 

In vivo experiment: before the onset of arthritis, rats were randomly assigned to six groups: Non, AA, GE (30, 60 and 120 mg/kg), and SIN (positive control group, 90 mg/kg) (n = 10 per group), in which AA rats were given intragastrically GE (30, 60 and 120 mg/kg) and SIN (positive control group, 90 mg/kg) from day 14 to 21 after immunizations. While in groups of normal and AA model, rats were given an equal volume of water at the same time. 

In vitro experiment: the rats were anesthetized and sacrificed on day 21 after immunization. FLS were cultured as described previously [16]. Groups were assigned as follows: Non group, AA group, GE group (25, 50 and 100 μg/mL), JNK inhibitor group (SP600125, 10 μM), ERK1/2 inhibitor group (PD98059, 10 μM), and p38MAPK inhibitor group (SB203580, 10 μM) (n = 3 per group).

### 4.4. Arthritis Assessment

Rats were examined every 3–4 days for signs of arthritis by two independent observers who were blinded to the experimental design. Non-injected hind paw volume was measured with MK-550 volume meter (Muromachi Kikai Co., Tokyo, Japan). The arthritic severity in each paw was graded on a scale of 0–4: 0, no swelling; 1, swelling of finger joints; 2, swelling of phalanx joint and digits; 3, swelling of the entire region down to the ankle; and 4, deformity or ankylosis. The maximum arthritis score was 12 including three secondary arthritis paws for each rat [25].

### 4.5. Histopathological Analysis

The rats were anesthetized and sacrificed on day 21 after immunization and synovial tissue of rats were collected under sterile conditions, fixed with 4% paraformaldehyde in PBS, and then embedded in paraffin for histopathological analysis. A series of paraffin sections (4 μm) were stained with hematoxylin and eosin (HE, 200×) and observed under a light microscope.

### 4.6. CCK8 Assay for FLS Viability

The viability of FLS was determined by the CCK8 assay. Cells were seeded into 96-well plates at 1 × 10^6^ cells/mL in 100 μL DMEM medium containing 10% FBS and pretreated with medium alone (control) or with GE at different concentrations (25, 50, 100 and 200 μg/mL). Then, 10 μL of CCK8 was added to each well and incubated for 48 h in an incubator at 37 °C with 5% CO_2_. The absorbance was tested at 540 nm in a microplate reader (Thermo Scientific Co., Waltham, MA, USA).

### 4.7. Proliferation Assay by MTT

FLS were cultured in triplicate in a concentration of 1 × 10^6^ cells/mL in 100 μL DMEM medium containing 10% FBS. First, the cells were stimulated with LPS (10 mg/L) for 48 h at 37 °C and 5% CO_2_. A 10 μL sample of MTT (5 mg/mL) was added before the end of stimulation for 4 h and then the cells were stimulated for 4 h continuously. After incubation, the cultures were centrifuged (760× *g*, 10 min) and all the supernatants were removed. A 150 μL of dimethysulfoxide (DMSO) was added to each well and the absorbance was examined at 570 nm using a MSS ELISA Microwell Reader (Thermo Scientific Co., Waltham, MA, USA).

### 4.8. Measurement of IFN-γ, IL-17, IL-4, and TGF-β1

Levels of IFN-γ, IL-17, IL-4, and TGF-β1 in FLS were evaluated by ELISA kits in accordance with the manufacturer’s instructions, respectively.

### 4.9. Western Blot Analysis

#### 4.9.1. Separation of Key Proteins from FLS In Vivo and In Vitro

Rats in each group were anaesthetized and sacrificed on day 21 after immunization. The fresh synovial tissues were taken out in sterile condition and put into culture dishes with D-Hank’s solution, washed and removed all of fat and connective tissue. Then, rats synovial tissues were cut into small pieces, placed in a clean glass homogenizer, to which was added a certain amount of RIPA lysate, homogenized on ice, and centrifuged (3000× *g*) at 4 °C. The supernatant was aspirated, to which was added a certain amount of 5× electrophoresis buffer, boiled in boiling water for 10 min, and preserved at −20 °C. FLS were washed with ice-cold PBS and lysed in cell lysis buffer. The proteins were separated by 10% sodium dodecyl-sulphate-polyacrylamide gel electrophoresis (SDS-PAGE).

#### 4.9.2. Western Blotting

The protein concentration was determined by the BCA method, and the proteins were electrophoretically transferred to polyvinylidene difluoride (PVDF) membranes (Millipore, Bedford, MA, USA). The membranes were incubated with blocking buffer (5% skim milk in Tris-buffered saline/Tween 20, TBST) at room temperature for 4 h on a rotary shaker. Primary antibodies specific for β-actin, JNK, p-JNK, ERK1/2, p-ERK1/2, p38, and p-p38 were incubated with the membranes overnight at 4 °C. Membranes were washed with TBST and incubated with the appropriate horseradish peroxidase (HRP)-conjugated goat anti-rabbit antibody for 2 h. Immunoreactive proteins were detected by enhancing chemiluminescence according to the manufacturer’s instructions (Pierce Biotechnology Inc., Waltham, MA, USA).

### 4.10. Statistical Analysis

Data were expressed as the means ± standard deviation (SD), in triplicate. Significant differences between groups were analysed by one-way ANOVA test and Student’s *t*-test. The results were considered statistically significant for *p* values less than 0.05.

## 5. Conclusions

Our study revealed that the inhibitory effect of GE on AA was associated with its ability to inhibit the hyperproliferation of FLS, down-regulated the production of IFN-γ and IL-17, up-regulated the level of IL-4 and TGF-β, and blocked the MAPK signalling pathway through inhibiting the phosphorylation of ERK1/2, JNK, and p38MAPK. The cross-talk among p38, ERK1/2, and JNK coordinate the balance of inflammatory and immune effects. Targeting MAPK signalling may be a new therapeutic strategy in inflammatory/autoimmune diseases. Further studies are still required to investigate its clinical relevance.
